# Influenza pandemic: perception of risk and individual precautions in a general population. Cross sectional study

**DOI:** 10.1186/1471-2458-7-48

**Published:** 2007-04-02

**Authors:** Ivar S Kristiansen, Peder A Halvorsen, Dorte Gyrd-Hansen

**Affiliations:** 1 Institute of Health Management and Health Economics, University of Oslo, Norway; 2 Institute of Public Health, University of Southern Denmark at Odense, Denmark; 3 Institute of Community Medicine, University of Tromsø, Norway

## Abstract

**Background:**

An influenza pandemic may have considerable impact on health and societal functioning. The aim of this study was to explore people's reflections on the consequences of a pandemic.

**Methods:**

Cross-sectional web-based survey of 1,168 Norwegians aged 16–82 years. The main outcome measures were answers to questions about a potential pandemic ("serious influenza epidemic"): statements about personal precautions including stockpiling Tamiflu^®^, the perceived number of fatalities, the perceived effects of Tamiflu^®^, the sources of information about influenza and trust in public information.

**Results:**

While 80% of the respondents stated that they would be "careful about personal hygiene", only a few would stay away from work (2%), or move to an isolated place (4%). While 27% of respondents were uncertain about the number of fatalities during an influenza pandemic, 48% thought it would be lower than the estimate of Norwegian health authorities (0.05%–1%) and only 3% higher. At least half of the respondents thought that Tamiflu^® ^might reduce the mortality risk, but less than 1% had personally purchased the drug. The great majority had received their information from the mass media, and only 9% directly from health authorities. Still the majority (65%) trusted information from the authorities, and only 9% reported overt distrust.

**Conclusion:**

In Norway, considerable proportions of people seem to consider the mortality risk during a pandemic less than health authorities do. Most people seem to be prepared to take some, but not especially disruptive, precautions.

## Background

Soon, a longer time will have elapsed since the last human influenza pandemic (1968) than between the previous two pandemics (1918 and 1957). During the last year, transmission of avian influenza to humans has been reported in several countries. Up until now there has been no evidence that avian influenza is able to spread among humans, but if this were to happen, a pandemic would be likely to develop. Experts at WHO believe that "the world is now closer to another influenza pandemic than at any time since 1968" [1], and the consequences may be considerable both in terms of fatalities and resource use [2,3]. The Norwegian Institute of Public Health states that people have "some reason to be scared" of a pandemic and indicates that 0.05%–1% of the population might die during an influenza pandemic [4]. This estimate is in accordance with the observed 0.65% in the US during the Spanish Flu (675,000 [5] out of a population of 103 millions [6]).

In 2005, to prepare for a pandemic, the Norwegian Government stockpiled oseltamivir (Tamiflu^®^) in quantities equivalent to 1.4 million courses of treatment for a total population of 4.6 millions. In general the possibilities of a pandemic have created considerable public concern, not least because of extensive media coverage of the global influenza situation and several reports of avian influenza in humans.

The current situation raises issues beyond the core public health challenges. First, what are people's sources of information about influenza pandemics, and do people trust the information they receive from public sources? If they do, health authorities will be better able to manage the consequences of a pandemic. Second, what are people's perceptions of the mortality risk during an influenza pandemic, and are they in line with the predictions from public health authorities? Third, what precautions might people take to protect themselves during a pandemic? If a considerable proportion of the population gives up normal activities such as work to protect themselves, this might disrupt social functions such as health care, policing and communication. The consequences may be devastating, at least in the short term, even if the death toll were moderate.

## Methods

TNS Gallup has developed an internet-based panel of about 25,000 Norwegians aged 15 years and over. The panel members have volunteered to respond to questionnaires via internet. E-mail alerts are sent when a questionnaire is ready to be filled in on the internet. The members receive bonus points each time they participate in a survey, and the points can be used to purchase household articles, *etc*. The response rate is typically 70–80%, and the survey is finalised when a pre-specified number of responses are obtained. For financial reasons, this study was restricted to 1,000 questionnaires among individuals aged 15–67 years of age, and 165 aged 68+. In total, 1,500 individuals aged 15–68 and 250 aged 68+ were invited, randomly chosen among the 25,000 panel members. In practice 1004+164 (78%) respondents filled in the questionnaire. The agreed number of responses was achieved earlier (3 days after the e-mail alert in early November 2005) than is usual for such surveys. The study was undertaken at a time when national and international mass media were very concerned about the pandemic threat [7].

The questionnaire (see appendix) posed eight questions about a potential "serious influenza outbreak": precautions during such an outbreak; the likely number of fatalities in Norway (*i.e*. the perceived risk in a population of 4.6 million); the number of avoided fatalities if Tamiflu^® ^were available to everybody (*i.e*. the absolute risk reduction, ARR); the maximum that respondents were willing to pay for a course of Tamiflu^® ^(paper accepted in Health Economics); whether they already had got hold of Tamiflu^®^; their sources of information about influenza; whether they trusted information provided by authorities; and self reported health. We chose to use the term "serious influenza outbreak" because we thought that many respondents might not be familiar with the term pandemic.

In the analysis of fatality data, we assumed that all fatalities and avoided fatalities would occur during a one year period. The respondents' perceived risks and Tamiflu^®^-effects can then be used to calculate one year mortality rates and absolute risk (incidence) reductions (ARR). The ARR was translated to numbers-needed-to-be-treated (NNT) by taking the reciprocal of ARR. Relative risk reduction from Tamiflu^® ^was calculated on the basis of mortality rates without Tamiflu^® ^and the respondents' reported ARR.

Information on age, sex, marital status, education, place of residence, and family income had been previously collected in 2005 for the whole panel. Along with self reported health and perceived risk, these variables were used as determinants of responses in multivariable logistic regression analyses. Data were analysed in SPSS and SAS.

## Results

The mean age of the respondents was 45 years (median 45, range 16–82) and 45% were female (Table 1). The respondents were fairly representative of the general population with respect to age, proportion of people living alone, and place of residence. Men and people with high income and education were somewhat overrepresented.

**Table 1:** Sociodemographic variables of lay people aged 16 – 82 (n = 1,168) responding to an internet survey about a serious influenza epidemic

		**Sample**	**Norwegian population**
**Age**^a^			
	15 – 29	21 %	23 %
	30 – 44	28 %	28 %
	45 – 59	29 %	25 %
	60 +	22 %	23 %
**Proportion female**^a^		45 %	51 %
**Proportion living alone**^b^		16 %	16 %
**Years of Education**^a^			
	0 – 10	14 %	21 %
	11 – 13	53 %	55 %
	14 +	33 %	24 %
**Region of Recidence**^a^			
	Central	51 %	50 %
	Southern/western	31 %	31 %
	Middle/northern	18 %	19 %
**Source of income**^c^			
	Work	61 %	64 %
	Pension (old age)	15 %	7 %
	National Insurance	7 %	10 %
	Other	17 %	19 %
**Household income**^a ^**(€1.00 = NOK8.00)**			
		€67,150	€57,188^d^

a) Compared to the Norwegian population aged 15+

b) Compared to the entire Norwegian population

c) Compared to the Norwegian population aged 16 – 74

d) Average household income in 2003 according to Statistics Norway

The majority of respondents had received information about influenza from the mass media (TV: 89%, newspapers: 85%, periodicals: 4%); a few directly from health authorities via internet or "influenza-phones" (Institute of Public Health: 2%, Food Safety Authority: 3%, GPs: 4%, Directorate of Health: 1%). Seventy-nine per cent of the respondents had received information from the mass media only. Sixty-five per cent trusted the information they had received directly or indirectly from authorities, while 9% distrusted it. In logistic regression, the odds for reporting overt distrust in or doubts about the information were greater among younger people (age < 45 years *vs *45+, OR 1.9, 95% CI 1.4 – 2.4), those with poor self-reported health (poorer than "very good" *vs *"very good", OR 1.5, 95% CI 1.2 – 2.0), those with poor education (<14 years *vs *14+, OR 1.4, 95% CI 1.1 – 1.8), those with a high perceived mortality risk (10,000+ deaths *vs *<10,000, OR 1.7, 95% CI 1.2 – 2.3) and people living in one specific region (south-west, OR 1.6, 95% CI 1.2 – 2.2 relative to the central region).

About one quarter of the respondents were uncertain about the mortality risk (number of fatalities) from a serious influenza epidemic, whereas the majority indicated it to be less than 1% (Table 2). For 48% of respondents the perceived risk was lower than predicted by the health authorities. Relative risk reduction (RRR) and the number needed to be treated (NNT) with Tamiflu^® ^were calculated except for respondents who were uncertain about the input numbers or indicated a greater number of fatalities with Tamiflu^® ^than without. Among respondents for whom we could calculate RRR and NNT, the vast majority of NNTs were above 1,000 and half of RRRs greater than 50% (Table 2). In total, only 12 (1%) of the respondents had purchased Tamiflu^®^.

**Table 2:** Lay people's anticipations of fatalities and effect of Tamiflu^® ^during a serious influenza epidemic^a^

**Absolute (baseline) risk (number of fatalities in a population of 4.6 mill)**	**Relative to the authorities' mortality prediction**^b^	**Proportion of respondents (n = 1168)**
0.0002% – 0.02%	Below	48 %
0.04% – 1.1%	Similar	22 %
2.2% – 22%	Above	3 %
Uncertain		27 %
**Relative risk reduction from Tamiflu**^®^		
< 50 %		24 %
50 – 100%		24 %
Inconsistent (ARR^b ^> baseline risk)		3 %
Uncertain		48 %
**Number needed to treat**		
< 1000		9 %
> 1000		41 %
Inconsistent (ARR^c^>baseline risk)		3 %
Uncertain		47 %

^a) ^Estimated from the respondents' anticipated number of fatalities and number of fatalities averted by Tamiflu^®^, relative to a total population of 4.6 million.

^b) ^The Norwegian Institute of Public Health states that 0.05%–1% of the population might die during an influenza pandemic

^c) ^ARR = absolute risk reduction

According to their responses, 80% would be "careful about personal hygiene", 9% would use face masks when outdoors, 2% would stay off work, 11% would stay at home and avoid contact with others, 4% would move to an isolated area (country side cottage, farm, *etc*.) while 16% would not take any special precautions if a serious influenza pandemic should break out. In logistic regression analysis, the odds for reporting isolation (*i.e*. stay home, stay off work or move to an isolated place) as a precaution were greater among those with poor self-reported health (poorer than "very good" *vs *"very good", OR 1.8, 95% CI 1.3 – 2.6) and those with high perceived fatality risk (10,000+ deaths *vs *<10,000, OR 3.2, 95% CI 2.2 – 4.8 compared to < 10,000 deaths).

## Discussion and conclusion

The results of this study indicate that most people consider influenza pandemic to be a public health threat, but about half of the respondents considered the mortality to be lower than predicted by the health authorities. Moreover, relatively few seem prepared to take precautions that would seriously disrupt their normal social functions. Few have purchased Tamiflu^® ^even though many consider it to be effective in preventing fatalities. The results, however, should be seen in terms of the limitations of the study. The study sample was not entirely representative of the adult Norwegian population in that women, poorly educated people, and those with lower incomes were somewhat underrepresented. Numeracy is a problem when asking people about fatality risk, but we asked about the number of fatalities instead of risk estimates because "natural frequencies" are known to be better understood than probabilities [8]. Still, 3% of the respondents indicated a greater number of avoided fatalities from using Tamiflu^® ^than the number without any treatment. When computing mortality rates, we assumed that the respondents estimated the number of fatalities or avoided fatalities for a Norwegian population of 4.6 million. To the extent that this assumption does not hold, our risk estimates are imprecise. While the responses to the question about Tamiflu^® ^purchase were based on actual behavior in present time, statements about precautions were based on intentions given thata pandemic hits Norway some time in the future. Consequently, the reported behavior during a pandemic may suffer from hypothetical bias. Such bias is likely since the majority of respondents will have limited or no information on the implications of a pandemic such as the Spanish Flu, and the lack of experience and information may clearly lead to respondents being rather uncertain about their true behaviour in such extraordinary circumstances.

That respondents were faced with only a few simple questions is a strength of the study. The rapid response to the questionnaire is an indication that people considered the issues to be important. The results seem germane to public health and crisis policy managers. First, the majority trusts the information they receive from health authorities even though many receive this information through mass media that tend to frighten or exaggerate in order to receive attention (less than 10% had sought information directly from health authorities websites or call systems). The highest selling newspaper wrote at one stage that about 25% of the population might die during a pandemic. In contrast, health authorities have indicated a mortality risk in the order of 0.05–1%, and about half of the respondents perceived the risk to be even lower than this. It is reassuring that an occasional "shock headline" seems to have little impact on people's risk perceptions, but also somewhat intriguing that the public in the light of extended media coverage have a tendency to underestimate the risk of mortality. Overall, it must be concluded that the media coverage has been balanced, or alternatively, the members of the public have effectively screened the flow of information that reached them. No more than about one in ten seems to be prepared to stay off work or move to isolated locations to protect themselves. Even though these results are based on hypothetical considerations, they indicate that few are prepared to take precautions that would disrupt vital functions of society. If authorities manage to avoid panic during a pandemic, the consequences may not be disastrous to social functioning unless fatality numbers get too high. It is worthwhile noting that the majority will take some precautions, and a pandemic will almost certainly have impact on travelling and other aspects of social and economic life. It seems contradictory when 11% said they would avoid contacts with others while only 2% would stay away off work. Presumably, most would go to work, but stay away from others the rest of the time. As many as 80% of the respondents would be more "careful about personal hygiene". Unfortunately, this term is imprecise and leaves us with little information on the exact precautions that the majority of respondents would be prepared to take. In a paper on non-pharmaceutical interventions for pandemic influenza, the World Health Organization states that there is limited evidence for an effect of face mask, "cough etiquette" or handwashing [9].

There are few similar studies reported. In a study of US public health workers, nearly half of them reported that they were likely not to report to duty during an influenza epidemic [10]. It should be noted that study location, type of respondents and type of survey was quite different.

Tamiflu^® ^is perceived by many as an effective drug against influenza fatalities, but few had got hold of the drug at the time of the survey (November 2005). Sales data indicate that about 1% of the Norwegian population has purchased Tamiflu^® ^during the subsequent 5 months. It should be noted, however, that health authorities and the Medical Association advised GPs against prescribing Tamiflu^® ^as a precaution except in special circumstances. A review questioned the effectiveness of antivirals for seasonal influenza, but the relevance of this in the context of influenza pandemic is unclear [11]. The government stockpiling of Tamiflu^® ^may have further reduced the perceived need to purchase it personally.

In a recent paper, the authors warn against a "pandemic of panic" [12]. Our results, however, indicate that only a minority of the population perceive the risk as greater than health authorities do, and most people indicate that they will take some, but not overly disruptive, precautions if a pandemic breaks out.

## Competing interests

The author(s) declare that they have no competing interests.

## Authors' contributions

All authors have contributed in designing the study and analysing the data. ISK drafted the paper, all authors have participated in revising the draft version, and all authors have read and accepted the final version.

## Appendix – The questionnaire

Q1: If a serious influenza epidemic should break out in Norway – what do you think you would do? (please, tick one or more options)

O No special measures

O Be careful about personal hygiene

O Use face mask outdoor

O Keep away from work

O Stay at home and avoid contact with others

O Go to a remote place like country cottage, farm or other

Q2: How many people would you think are going to die in Norway if a serious influenza breaks out? (please, tick one option)

O 10

O 50

O 100

O 250

O 500

O 1.000

O 2.000

O 4.000

O 7.000

O 10.000

O 25.000

O 50.000

O 100.000

O 250.000

O 500.000

O 1.000.000

O Uncertain

Q 3: How many fatalities do you think could be avoided if the drug Tamiflu were available to everybody? (please, tick one option)

O 10

O 50

O 100

O 250

O 500

O 1,000

O 2,000

O 4,000

O 7,000

O 10,000

O 25,000

O 50,000

O 100,000

O 250,000

O 500,000

O 1,000,000

O Uncertain

Q4: How much would you at *maximum *be willing to pay in order to have a course of Tamiflu drug available in case you would need it? (please, tick one option)(€1.00 = NOK8.00)

O Less than NOK 50

O NOK 50

O NOK 100

O NOK 250

O NOK 500

O NOK 1.000

O NOK 1.500

O NOK 2.000

O NOK 3.000

O NOK 5.000

O NOK 7.500

O NOK 10.000

O More than NOK 10.000

Q5. Have you got hold of Tamiflu to yourself or your closest family members?

O Yes

O No

Q6. How is your health status? (please, tic kone option)

O Poor

O Not so good

O Quite good

O Very good

Q7. From what sources have you received information about influenza? (please, tic kone or more options)

O Newpapers

O Weekly magazines

O TV

O The website of the National Public Health Institute

O The "influenza-phone" of the National Public Health Institute

O The website of the Directorate for Health and Social Affairs

O The Norwegian Food Safety Authority

O Your general practitioner

O Other

Q8. Do you trust the information provided by authorities about influenza-situation?

O Yes

O No

O Uncertain

## Pre-publication history

The pre-publication history for this paper can be accessed here:


